# Assessing the Impact of Inflammation on Erythropoietin Resistance in Hemodialysis: The Role of the NLR

**DOI:** 10.3390/jcm14103411

**Published:** 2025-05-13

**Authors:** Caterina Carollo, Alessandra Sorce, Ettore Mancia, Emanuele Cirafici, Maria Elena Ciuppa, Benedetto De Biasio, Giuseppe Mulè, Giuliano Brunori

**Affiliations:** 1 Unit of Nephrology and Dialysis, Hypertension Excellence Centre, Department of Health Promotion, Mother and Child Care, Internal Medicine and Medical Specialties (PROMISE), University of Palermo, 90133 Palermo, PA, Italyemanuele.cirafici@community.unipa.it (E.C.); benedetto.debiasio@policlinico.pa.it (B.D.B.); giuseppe.mule@unipa.it (G.M.); 2 Nephrology and Dialysis, Santa Chiara Regional Hospital, APSS Trento, 38123 Trento, TN, Italy; ettore.mancia@apss.tn.it (E.M.); giuliano.brunori@apss.tn.it (G.B.); 3 Department of Health Promotion, Mother and Child Care, Internal Medicine and Medical Specialties, University of Palermo, 90133 Palermo, PA, Italy; mariaelena.ciuppa@community.unipa.it

**Keywords:** NLR, hemodialysis, inflammation, erythropoietin resistance index, CKD

## Abstract

**Background:** The neutrophil-to-lymphocyte ratio (NLR) has emerged as a readily available marker of systemic inflammation and immune dysregulation. In patients undergoing hemodialysis, inflammation is a known contributor to erythropoietin resistance. However, the relationship between the NLR and the erythropoietin resistance index (ERI) has not been extensively characterized. **Methods:** A total of 317 hemodialysis patients were retrospectively evaluated and stratified into tertiles based on NLR levels. Biochemical, inflammatory, and anthropometric variables were compared across groups. Spearman’s rank correlation was used to assess the relationship between the NLR and the ERI. Receiver operating characteristic (ROC) curve analysis was performed to evaluate the predictive ability of the NLR for erythropoietin resistance, defined as ERI > 10. Subsequently, linear and logistic regression models were employed to examine the independent association between the NLR and the ERI, adjusting for relevant covariates. **Results:** Higher NLR tertiles were significantly associated with increased CRP, lower serum iron, and elevated ERI (*p* = 0.002). Spearman’s analysis revealed a modest but significant correlation between the NLR and the ERI (ρ = 0.31, *p* < 0.0001). ROC analysis identified an NLR threshold of 4.4 for detecting ERI > 10. In multivariable analysis, the NLR was independently associated with the ERI both as a continuous variable (β = 0.848, *p* = 0.046) and as a binary outcome (OR = 1.22, 95% CI: 0.95–1.24, *p* = 0.0021), while serum iron and hemoglobin also emerged as significant predictors. **Conclusions:** In this cohort of hemodialysis patients, a higher NLR was independently associated with increased erythropoietin resistance, suggesting its potential utility as an accessible inflammatory biomarker in anemia management. These findings add to the limited but growing body of evidence supporting the prognostic role of the NLR in dialysis populations and warrant further validation in prospective studies.

## 1. Introduction

Anemia is a prevalent and burdensome complication in patients with end-stage renal disease (ESRD) undergoing maintenance hemodialysis. Its etiology is multifactorial and includes reduced erythropoietin production, iron deficiency, chronic inflammation, and shortened red blood cell lifespan [1,2,3]. The management of anemia in hemodialysis patients predominantly relies on the use of erythropoiesis-stimulating agents (ESAs), which have significantly improved outcomes for many [4]. However, a considerable subset of patients remains hyporesponsive to ESAs, requiring progressively higher doses to maintain target hemoglobin levels. This phenomenon, referred to as erythropoietin resistance, is associated with increased morbidity, cardiovascular events, and all-cause mortality [5,6].

Epidemiological studies indicate that approximately 10–15% of patients undergoing chronic dialysis experience clinically significant resistance to ESAs, leading to higher treatment costs, prolonged hospitalization, and poorer long-term outcomes [7]. The erythropoietin resistance index (ERI), commonly used to quantify ESA responsiveness, is calculated as the weekly weight-adjusted dose of erythropoietin divided by the hemoglobin concentration. Typically, an ERI above 10 IU/kg/week/gHb is considered indicative of resistance, although the threshold may vary across studies. Elevated ERI levels are frequently observed in patients with persistent inflammation, nutritional deficiencies, and other comorbidities [8,9,10].

Inflammation is a well-established contributor to ESA hyporesponsiveness [11,12]. Chronic low-grade inflammation in patients with ESRD can impair erythropoiesis through various mechanisms, including inhibition of erythropoietin signaling, suppression of erythroid progenitor cell proliferation, and induction of hepatic hepcidin production. Increased hepcidin levels reduce iron bioavailability by inhibiting iron absorption from the gut and promoting iron sequestration in macrophages [13]. In addition to inflammation, protein–energy wasting (PEW) is another critical factor affecting erythropoietin responsiveness and is highly prevalent in hemodialysis patients. PEW, which involves a depletion of both protein and energy stores, often correlates with markers of inflammation, malnutrition, and poor clinical outcomes [14]. Studies have shown that PEW is associated with increased mortality, cardiovascular events, and resistance to ESA therapy, highlighting the intertwined relationship between nutritional status, inflammation, and anemia management [15,16].

Despite its significance, the management of both anemia and PEW in hemodialysis patients remains challenging. Identifying reliable, easily accessible biomarkers that can reflect systemic inflammation and predict ESA resistance is crucial for improving patient outcomes. The neutrophil-to-lymphocyte ratio (NLR), a simple and cost-effective inflammatory marker derived from a routine complete blood count, has recently gained attention as a potential tool for this purpose. The NLR reflects the balance between the innate (neutrophil-mediated) and adaptive (lymphocyte-mediated) immune responses, and has been linked to adverse clinical outcomes in a variety of conditions, including cardiovascular diseases, malignancies, and chronic kidney disease [17,18,19]. One of its key advantages is its ability to provide a snapshot of low-grade inflammation, which is often undetectable using traditional inflammatory markers such as C-reactive protein (CRP) [20].

Recent studies have explored the association between various inflammatory markers and ESA hyporesponsiveness, including CRP, ferritin, and interleukins [21,22,23]. However, data specifically linking the NLR with erythropoietin resistance are limited and inconsistent. Furthermore, while the majority of studies focus on small, heterogeneous cohorts, few have examined the prognostic significance of the NLR in predicting adverse outcomes in patients with ESRD, including all-cause mortality, hospitalization, or cardiovascular events.

In a previous study conducted by our group, we identified a significant association between higher NLR values and increased mortality in HD patients, with a notable correlation between the NLR and the ERI [18]. Building on these findings, the present study aims to further investigate the link between systemic inflammation, as measured by the NLR, and erythropoietin resistance. We hypothesize that an elevated NLR is independently associated with higher ERI values and could serve as a practical marker to stratify patients at risk of ESA hyporesponsiveness.

The findings from this study may ultimately guide clinicians in using the NLR as a practical, cost-effective tool to predict both ESA resistance and patient prognosis, potentially leading to more tailored and efficient management of anemia and overall care in hemodialysis patients.

## 2. Materials and Methods

**Figure 1:**
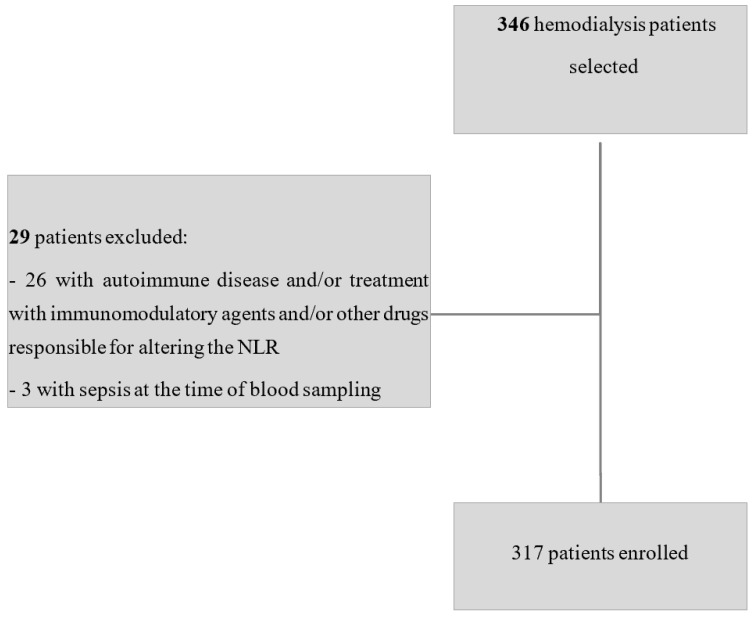
Flowchart of sample selection.

This multicentric retrospective observational study was conducted on hemodialysis patients from centers affiliated with the Nephrology and Multizonal Dialysis Unit at Santa Chiara Hospital in Trento. Between 1 January 2020 and 31 August 2022, 346 patients were identified as eligible for enrollment. After applying the study’s inclusion and exclusion criteria, 317 patients were ultimately recruited (Figure 1). All patients were adults (≥18 years) undergoing chronic hemodialysis for at least three months, a duration chosen to ensure clinical stability and to minimize the impact of acute changes on laboratory parameters. In addition, patients were included only if complete demographic, clinical, and laboratory data were available, including those necessary for calculating the NLR, as well as related inflammatory indices such as the platelet-to-lymphocyte ratio (PLR) and derived NLR (d-NLR), and for computing the ERI.

Patients were excluded at admission if they were receiving pharmacological regimens known to alter leukocyte counts (for example, antibiotics, immunoglobulins, monoclonal antibodies, or corticosteroid therapy at doses exceeding 20 mg), or if they had a history (recent or remote) of conditions capable of significantly affecting the leukocyte formula, such as hematologic malignancies, autoimmune diseases, or chronic inflammatory disorders. Subjects with ongoing sepsis at the time of blood sampling or with any acute conditions (for instance, patients who had received blood transfusions in the preceding three months) were also excluded in order to mitigate potential confounding effects on inflammatory markers.

Data were retrospectively collected from electronic medical records via the TSS^®^ (Therapy Support Suite, version 1.8.05, Fresenius Medical Care, Bad Homburg, Germany) management software. For each patient, information regarding demographics, medical history, clinical characteristics, and laboratory results was extracted. Routine biochemical parameters were measured using standard techniques on autoanalyzers at baseline.

The complete blood count was used to calculate the inflammatory markers: the NLR was determined by dividing the number of neutrophils by the number of lymphocytes, the PLR by dividing the platelet count by the number of lymphocytes, and the d-NLR by dividing the neutrophil count by the difference between the total leukocyte count and the number of neutrophils. Additionally, the dose of erythropoietin administered weekly, normalized to body weight and hemoglobin concentration, was used to calculate the ERI using the following formula:

ERI = Weekly Erythropoietin Dose (UI)/Body Weight (kg) × Hemoglobin (g/dL)

Erythropoietin resistance was defined as an ERI value greater than 10 IU/week/kg/g/dL. This threshold aligns with values reported in previous studies, where mean ERI values ranged approximately between 8 and 14 IU/week/kg/g/dL [24,25,26,27].

Due to the retrospective nature of this study, all patient data were de-identified prior to statistical analysis, in accordance with applicable privacy regulations Therefore, ethical approval from the local ethics committee was not required. All procedures followed were in accordance with the ethical standards of the institution and the Declaration of Helsinki.

### Statistical Analysis

Statistical analysis was performed using SPSS (version 26, IBM Corp, Armonk, NY, USA) and R (version 4.0.2). Descriptive statistics were computed for all variables, and the distribution of continuous variables was assessed using histograms and the Shapiro–Wilk test. Normally distributed data were expressed as mean ± standard deviation (SD), while non-normally distributed data were expressed as median (interquartile range, IQR). Categorical variables were summarized as frequencies and percentages.

To assess the relationship between the neutrophil-to-lymphocyte ratio (NLR) and erythropoietin resistance index (ERI), the patients were stratified into tertiles based on NLR and ERI values. This stratification allowed for the evaluation of potential differences in clinical characteristics and outcomes between the low, intermediate, and high groups.

Differences between these tertiles were evaluated using a chi-squared test with Fisher’s correction for categorical variables, one-way ANOVA for normally distributed continuous variables, and the Kruskal–Wallis test for continuous variables with non-Gaussian distribution.

Linear regression analysis was conducted to assess the relationship between the ERI and the independent variables.

Subsequently, interaction terms were introduced into the multivariable model to explore potential effect modification between the independent variables. Interaction terms were tested between the NLR and the following covariates: age, sex, diabetes status, serum albumin levels, iron, hemoglobin, and C-reactive protein (CRP). These interactions were included in the multivariable models to assess their potential effect on erythropoietin resistance. The significance of each interaction term was evaluated based on the *p*-value, with a threshold for statistical significance set at 0.05.

Also multivariate logistic regression was performed to examine the association between the NLR and the ERI with clinical outcomes (e.g., hemoglobin levels, C-reactive protein, albumin levels, and other laboratory parameters). In the logistic regression model, ERI was analyzed as a dichotomous dependent variable using the 10-point cut-off.

The Spearman rank correlation was used to assess the strength of the association between the NLR and the ERI.

A *p*-value < 0.05 was considered statistically significant for all analyses. All tests were two-tailed.

## 3. Results

A total of 317 participants were stratified into tertiles based on their NLR, and key biochemical and anthropometric variables were compared across groups (Table 1).

A trend toward increasing age was observed across tertiles, with a borderline significant difference (*p* = 0.0504), while no significant differences were observed in weight (*p* = 0.764) or sex distribution. Dialysis vintage increased significantly with higher NLR tertiles (*p* = 0.003).

Among inflammatory markers, CRP levels were significantly higher in the upper tertiles (*p* = 0.0002). Both platelet-to-lymphocyte ratio (PLR) and derived NLR (d-NLR) also increased progressively and significantly (*p* < 0.000001 for both). Platelet count did not differ significantly across groups (*p* = 0.672).

Regarding biochemical markers related to nutritional and metabolic status, no significant differences were found for total proteins (*p* = 0.605), albumin (*p* = 0.094), total cholesterol (*p* = 0.896), plasma urea (*p* = 0.734), potassium (*p* = 0.184), or phosphorus (*p* = 0.646). Serum iron (*p* = 0.0005) and transferrin (*p* = 0.006) levels were significantly lower in higher NLR tertiles. A non-significant trend toward higher blood glucose values was noted (*p* = 0.150).

Finally, ERI values showed a significant increase across NLR tertiles (*p* = 0.002).

**Figure 2:**
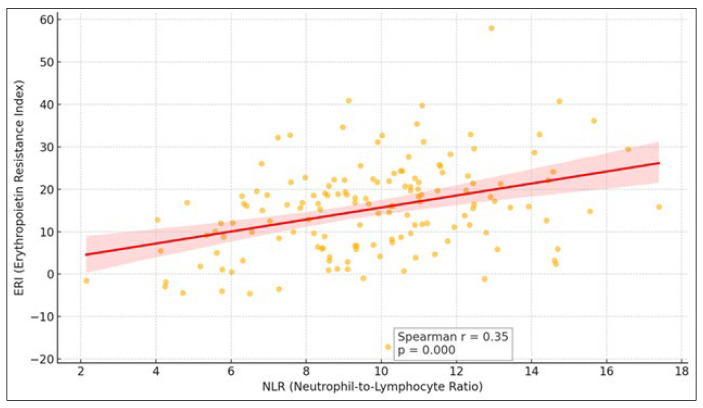
Spearman’s correlation between NLR and ERI. The regression line indicates the general trend in the relationship between the two variables. The shaded area around the regression line represents the 95% confidence interval for the estimated line.

A Spearman’s rank correlation was performed to evaluate the relationship between the NLR and the ERI. The analysis revealed a modest but statistically significant positive correlation, suggesting that higher NLR values may be modestly associated with increased erythropoietin resistance (Figure 2).

**Figure 3:**
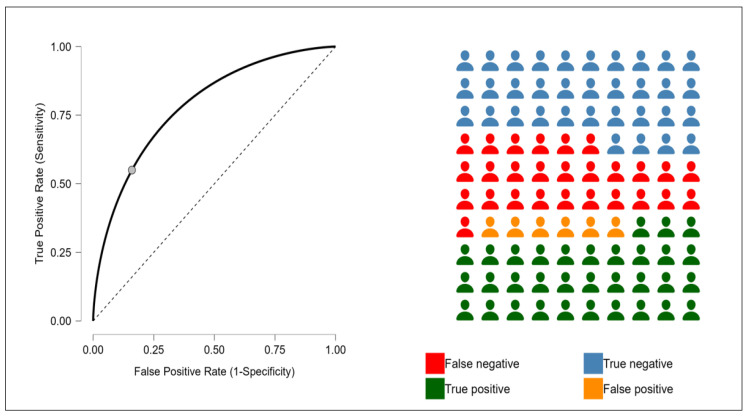
The figure evaluates a binary classification model, featuring an ROC curve to show predictive performance and a confusion matrix highlighting classification outcomes.

Following the Spearman’s rank correlation analysis, a receiver operating characteristic (ROC) curve analysis was performed to further assess the predictive ability of the NLR for identifying erythropoietin resistance. In this analysis, the outcome variable was defined as an ERI value greater than 10, which has been previously identified as the threshold for erythropoietin resistance in clinical settings (Figure 3).

The optimal cut-off value for the NLR was determined using the Youden Index, which maximizes the sum of sensitivity and specificity to identify the threshold that best discriminates between erythropoietin-resistant (ERI > 10) and non-resistant patients. The analysis identified an NLR value of 4.4 as the optimal cut-off point. The ROC analysis demonstrated a fair diagnostic performance of the NLR in predicting erythropoietin resistance (ERI > 10). Sensitivity was moderate at 0.550, indicating that the model correctly identified 55% of resistant patients. More notably, specificity was high (0.840), reflecting a strong ability to correctly exclude non-resistant individuals. This combination suggests that the test is particularly effective in confirming the absence of resistance when the NLR is low. The positive predictive value (PPV = 0.838) is a clear strength, indicating that when the NLR predicts resistance, it is correct over 83% of the time. The low false discovery rate (FDR = 0.162) suggests a low probability of overestimating resistance.

To further explore the association between the NLR and erythropoietin resistance, two regression models were applied. A linear regression was first performed using the ERI as a continuous dependent variable, to assess the trend between increasing NLR and ERI values (Table 2). Subsequently, a logistic regression was conducted using the ERI as a binary outcome (cut-off > 10) to evaluate the odds of erythropoietin resistance associated with the NLR (Table 3). This dual approach allowed for both a detailed and a clinically oriented interpretation of the relationship.

The NLR, iron, and hemoglobin were found to be independently associated with the ERI. These factors remained significant in the model; conversely, sex, age, comorbidities, electrolyte levels, cholesterol, protein levels, albumin, and CRP were excluded from the final model, as they did not demonstrate a statistically significant association with the ERI after adjusting for other variables. In order to further assess the potential for complex relationships among the variables, we also examined possible interaction effects between the NLR, iron, and hemoglobin in the context of the ERI. Specifically, interaction terms were added to the regression model to explore whether the relationship between each variable and the ERI was modified by the presence of the others. However, after adjusting for all relevant factors, none of the interaction terms were found to be statistically significant. This suggests that the associations between the NLR, iron, hemoglobin, and the ERI are independent.

In the multivariable logistic regression model assessing predictors of erythropoietin resistance (defined as ERI > 10), the NLR was significantly associated with the outcome. Specifically, higher NLR values were associated with increased odds of resistance (OR: 1.22, 95% CI: 0.95–1.24, *p* = 0.0021). Serum iron showed an inverse association with the outcome (OR: 0.77, 95% CI: 0.99–1.42, *p* = 0.0035), suggesting that lower iron levels may contribute to reduced responsiveness to erythropoietin. Variables such as sex, age, hemoglobin, comorbidities, CRP, albumin, and blood glucose were not retained in the final model due to lack of statistical significance.

## 4. Discussion

In this study, we explored the association between the NLR and a range of biochemical, inflammatory, and nutritional markers in a cohort of patients undergoing maintenance hemodialysis. By stratifying participants according to NLR tertiles, we aimed to investigate the extent to which the NLR reflects underlying inflammatory burden, alterations in iron metabolism, and potential immune dysregulation.

Resistance or reduced responsiveness to ESAs can arise from various factors. While iron deficiency is the primary cause, some ESRD patients continue to show resistance to ESAs despite receiving adequate iron supplementation. Other factors that can contribute to ESA resistance or hyporesponsiveness in ESRD patients with sufficient iron levels include ongoing inflammation, chronic blood loss, inadequate dialysis, hyperparathyroidism, aluminum toxicity, vitamin deficiencies, malnutrition, testosterone deficiency, and certain cancers [28].

The NLR has emerged as a simple, cost-effective biomarker reflecting systemic inflammation and immune dysregulation. Originally studied in oncology, the NLR has since gained relevance across various clinical settings, including cardiovascular disease, chronic kidney disease (CKD), and critical illness [29,30,31,32]. In the context of CKD and hemodialysis, an elevated NLR has been associated with increased mortality, cardiovascular events, and poor nutritional status [18,33,34]. Its prognostic value is thought to derive from its ability to capture the dual impact of neutrophil-driven inflammation and lymphocyte-mediated immune suppression. Despite its increasing clinical use, the utility of the NLR in predicting specific outcomes—such as erythropoietin resistance—remains underexplored, with only a limited number of studies providing preliminary evidence of its role in this domain.

Interestingly, while the NLR and the PLR showed statistically significant increases across tertiles, platelet and neutrophil counts remained stable, suggesting that the differences in these ratios were primarily driven by a reduction in lymphocyte count. This pattern points toward a state of lymphopenia, which is commonly observed in dialysis patients and reflects both immune system exhaustion and chronic inflammation. Lymphopenia has clinical implications beyond immune suppression—it may amplify the pro-inflammatory signal by reducing the regulatory and anti-inflammatory lymphocytic activity [35,36].

This inflammatory and immunological imbalance appears to directly impact erythropoietin responsiveness, as shown by the significant increase in the ERI across NLR tertiles. This finding supports the hypothesis that an elevated NLR is not merely a passive marker of inflammation but may reflect active mechanisms contributing to ESA hyporesponsiveness. Inflammatory cytokines such as IL-6 and TNF-α are known to impair erythroid progenitor proliferation and iron availability via hepcidin-mediated pathways, mechanisms that are consistent with the observed reductions in serum iron and transferrin in higher NLR groups [37,38].

IL-6 plays a pivotal role in the inflammatory cascade and has been extensively implicated in the pathogenesis of erythropoietin resistance in patients undergoing hemodialysis. As a pro-inflammatory cytokine, IL-6 contributes to the development of anemia by inhibiting erythropoiesis and promoting iron sequestration through the induction of hepcidin production. Elevated IL-6 levels have been associated with higher ERI values and a poor response to ESA therapy in multiple studies [16,39], emphasizing its clinical significance. While IL-6 was not directly measured in our cohort, the NLR has been previously correlated with IL-6 levels [40,41], suggesting it may serve as an indirect marker of IL-6-driven immune activation. However, this relationship requires further investigation, and future studies measuring IL-6 directly would help clarify the potential connection between the NLR and IL-6 in this context. The observed association between a high NLR and elevated ERI values likely reflects an underlying IL-6-mediated inflammatory state, supporting the potential use of the NLR as a surrogate marker for cytokine-induced ESA hyporesponsiveness. This relationship is further validated by recent findings, which suggest that IL-6 plays a critical role in modulating iron homeostasis and erythropoiesis, thereby influencing ESA efficacy in chronic dialysis patients [42].

Moreover, the association between longer dialysis vintage and higher NLR tertiles suggests a cumulative inflammatory burden over time. This chronic exposure may further exacerbate both immune dysfunction (as indicated by lymphopenia) and anemia management challenges.

The observed association between the NLR and the ERI in our study is consistent with the few available reports in the literature. For instance, Zhang et al. demonstrated that higher NLR levels were significantly associated with increased ERI values, suggesting that systemic inflammation plays a central role in modulating erythropoietin responsiveness in hemodialysis patients. This study of 299 hemodialysis patients aimed to investigate the relationship between the NLR, the PLR, and EPO responsiveness, measured by the ERI. Multivariate linear regression revealed that only the NLR (β = 0.13, *p* = 0.024) was significantly associated with a higher ERI, indicating worse EPO responsiveness [43]. Similarly, Valga et al. in a multicenter cross-sectional study of 397 hemodialysis patients, they found both the NLR and the PLR were significantly associated with the ERI, with NLR (*p* < 0.0001) and PLR (*p* < 0.0001) levels being significant predictors in regression analysis. The AUC for the PLR predicting erythropoietin resistance was 0.681, with a PLR cut-off of 125.5 showing a sensitivity of 80.95%, demonstrating slightly better predictive performance than the NLR [44].

Conversely, Pineault et colleagues found a significant positive correlation between NLR and CRP levels (r = 0.45, *p* < 0.001), indicating that an elevated NLR reflects increased systemic inflammation. Additionally, the NLR was inversely correlated with serum albumin levels (r = −0.47, *p* < 0.001), suggesting that a higher NLR is associated with poorer nutritional status. However, no significant correlation was observed between the NLR and the ERI (r = 0.32, *p* = 0.32), implying that the NLR may not be a reliable marker for ESA resistance in their population [45].

In interpreting our findings, it is important to consider the potential role of PEW, a common and clinically significant condition in ESRD patients undergoing hemodialysis [46]. PEW, characterized by the loss of body protein mass and energy reserves due to a combination of inadequate dietary intake, chronic inflammation, metabolic derangements, and the catabolic effects of dialysis, is known to contribute to adverse outcomes including ERI values and increased mortality [14,47,48]. Although in our cohort classical nutritional markers such as serum albumin, total protein, and cholesterol did not show significant differences across NLR tertiles, the observed reduction in serum iron and transferrin levels in patients with higher NLR values may reflect an early or subclinical catabolic state compatible with PEW. This is particularly relevant given the well-documented interaction between inflammation and malnutrition in driving ESA hyporesponsiveness. Prior studies have demonstrated that PEW is independently associated with elevated ERI values, and that composite indices such as the malnutrition–inflammation score (MIS) are superior to individual biochemical parameters in predicting ESA resistance and clinical outcomes [49,50].

In this context, the NLR—while primarily an inflammatory biomarker—may also act as a surrogate indicator of broader immune-nutritional dysregulation, encompassing both lymphopenia and neutrophilia, which are commonly observed in PEW. The association between a high NLR and increased ERI values observed in our study may therefore, at least in part, reflect the combined effects of inflammation and early-stage malnutrition. Importantly, while albumin levels were not significantly different across groups, it is recognized that albumin alone lacks sensitivity for detecting early or moderate PEW, especially in the presence of inflammation. Our findings support the hypothesis that inflammation-mediated PEW contributes to erythropoietin resistance and suggest that simple and accessible markers such as the NLR may serve as valuable screening tools in this context. Our results can contribute novel insights into how inflammatory markers, particularly the NLR, might influence ESA responsiveness, potentially leading to more tailored treatment approaches for patients with erythropoietin resistance.

This study has some limitations. First, being retrospective, it cannot establish causal relationships and is subject to potential biases, such as selection and information bias. The heterogeneity of the population in terms of demographics limits the generalizability of the results. Additionally, the use of stepwise regression may have excluded clinically relevant variables due to their lack of statistical significance. Lastly, the absence of data on dietary habits, and other inflammatory markers like IL-6, a key inflammatory marker, limited our ability to fully explore its role in erythropoietin resistance. Further research should aim to integrate body composition analysis, dietary assessments, and comprehensive inflammation–nutrition scoring systems to better characterize the interplay between PEW, inflammation, and ESA responsiveness. Interventions targeting both nutritional support and inflammatory control may offer a path toward improved anemia management and overall outcomes in this vulnerable patient population.

In summary, elevated NLR values in this hemodialysis population appear to capture a multifaceted risk profile: chronic inflammation, impaired iron metabolism, erythropoietin resistance, and lymphocyte depletion—all of which are interconnected and clinically relevant to patient outcomes.

## 5. Conclusions

This study provides novel insights into the relationship between systemic inflammation, as measured by the NLR, and erythropoietin resistance in hemodialysis patients. We observed that higher NLR values are independently associated with ERI levels, both as a continuous and dichotomous outcome. These findings suggest that the NLR, a simple and widely available marker of inflammatory status, may serve as a useful tool for identifying patients at higher risk of hyporesponsiveness to ESAs.

Importantly, our results align with and expand upon the limited existing literature, which has suggested a potential role for the NLR in anemia management in chronic kidney disease. While the underlying mechanisms remain multifactorial—including the influence of iron metabolism, nutritional status, and inflammatory cytokines such as IL-6—our data support the integration of the NLR into broader predictive models for ESA responsiveness.

Further investigation into the pathophysiological pathways linking inflammation and erythropoietin resistance—particularly those related to the malnutrition–inflammation complex and PEW syndrome—may provide new therapeutic targets to optimize anemia control in this vulnerable population.

## Figures and Tables

**Table 1:** Biochemical, metabolic, and anthropometric profiles across neutrophil-to-lymphocyte ratio (NLR) tertiles.

	1° TERTILE n = 105	2° TERTILE n = 106	3° TERTILE n = 106	p
Age, years	68 (56–76)	71 (61–80)	73 (61–81)	0.0504
Male sex, n (%)	75 (71)	78 (74)	76 (72)	0.1010
Weight, kg	68 (59–79)	67 (58–77)	66 (57–78)	0.7641
Dialysis vintage, months	33 (16–46)	37 (17–68)	42 (23–71)	0.0030
ERI	13 (6–13)	15 (13–18)	20 (17–23)	0.0022
PLT, 10^9^/L	200 (148–258)	209 (160–253)	202 (154–257)	0.672
CRP, mg/dL	2.7 (1.9–7.6)	3.9 (2.2–10.2)	9.3 (3.3–25)	0.0002
PLR	108 (87–140)	155 (125–206)	239 (190–324)	<0.000001
Neutrophils, 10^9^/L	4300 (3900–4600)	4300 (4100–4700)	4500 (4200–4800)	0.4233
Lymphocyte, 10^9^/L	1840 (1380–2200)	1330 (1000–1645)	845 (660–1100)	<0.000001
d-NLR	1.29 (0.99–1.47)	2.1 (1.8–2.4)	3.4 (3–4.1)	<0.000001
Total proteins, g/dL	6.5 (6.2–6.8)	6.5 (6.2–6.9)	6.5 (6.1–6.8)	0.6050
Albumin, g/L	32 (29–36)	33 (31–35)	32 (28–35)	0.0944
Serum iron, µg/dL	51 (37–70)	48 (38–60)	39 (29–52)	0.0005
Transferrin, mg/dL	184 (160–200)	184 (160–210)	170 (130–190)	0.0067
Total cholesterol, mg/dL	147 (127–168)	151 (124–178)	149 (123–175)	0.8962
Plasma urea, mg/dL	156 ± 40	153 ± 38	150 ± 45	0.7341
Potassium, mmol/L	5 ± 0.7	5.1 ± 0.8	5.1 ± 0.9	0.1843
Phosporus, mg/dL	5.3 (4.4–6.3)	5.1 (4.1–6.5)	5.1 (4.4–6.3)	0.6466
Blood glucose, mg/dL	116 (97–139)	121 (97–150)	125 (104–157)	0.1500

CRP: C-reactive protein, PLR: platelet-lymphocyte ratio, d-NLR: derived neutrophil-lymphocyte ratio, ERI: erythropoietin resistance index.

**Table 2:** Independent correlates of ERI in multiple linear regression analysis.

Model		Unstandardized	Standard Error	Standardized	t	*p*
M₁	(Intercept)	21.133	5.493		3.847	<0.001
	Hemoglobin	−0.067	0.069	−0.061	−0.965	0.02
	NLR	0.848	0.423	0.124	2.004	0.046
	Serum Iron	−0.003	0.004	−0.057	−0.887	0.001

**Table 3:** Independent correlates of ERI in multiple logistic regression analysis.

	Unstandardized	SE	*p*	Exp(b)	95% CI of Exp(b)
Serum Iron	−0.004489	0.003810	0.0035	0.7710	0.9882 to 1.4229
NLR	0.01964	0.03415	0.0021	1.2198	0.9541 to 1.2412

## Data Availability

The data presented in this study are available on request from the corresponding author.
